# A new technique for trans-perirectal iodine-125 seed implantation in prostatic cancer guided by CT and 3D printed template: Two case reports

**DOI:** 10.3389/fonc.2022.1031970

**Published:** 2022-10-21

**Authors:** Xuemin Di, Hongtao Zhang, Xiaoli Liu, Jinxin Zhao, Zhen Gao, Huimin Yu, Xiaohua Su, Yansong Liang, Juan Wang

**Affiliations:** Department of Oncology, Hebei General Hospital, Shijiazhuang, China

**Keywords:** prostate cancer, brachytherapy, 3D-printed template, iodine-125 seed implantation, CT guidance, low-dose-rate brachytherapy

## Abstract

Low-dose-rate prostate brachytherapy with permanent iodine-125 is an important curative treatment for low-risk prostate cancer, and it has been demonstrated that brachytherapy with permanent seeds is an effective treatment. However, differences in prostate volume, spatial location, and gland deformation between images obtained in the pre-planning phase and those obtained during the implantation procedure affect accurate delivery of the pre-planned dose. Furthermore, the complicated procedure could be a burden to elderly patients, for example, the risks associated with general anesthesia. In addition, ultrasound images are not as clear as computed tomography (CT) images with regard to identifying the location of seeds. Therefore, a new method for guidance during the procedure is urgently needed. Here, we have described a new method for precise trans-perirectal insertion of radioactive iodine-125 seeds in patients with prostate cancer under the guidance of CT and a 3D-printed template. These are some of the advantages of this technique over the standard procedure for seed implantation in the prostate: It requires only local anesthesia, the pre-planning phase can be completed before the procedure, and the operation time is considerably shorter. This report describes trans-pararectal iodine-125 seed brachytherapy for prostate cancer under local anesthesia and the guidance of a 3D printed template in two elderly patients. The dose parameters determined in the preoperative planning phase were verified postoperatively and found to be consistent. Further, the procedure was completely successfully with no major complications in both cases, and the patients’ prostate-specific antigen levels were normal at the most recent follow-up conducted 50 months after the procedure. Therefore, this technique seems promising for prostate cancer brachytherapy, and its application needs to be researched and extended further in the future.

## Introduction

Brachytherapy for prostate cancer has rapidly been gaining popularity in the USA (1–4) as an effective and safe treatment for localized prostate cancer. In one study that examined treatment for early-stage prostate cancer in the USA, brachytherapy was found to be a component of treatment in 36% of the cases (5). In 1917, Barringer introduced interstitial implantation of radiation needles in patients with prostatic cancer as a new therapeutic modality (6). The needles were inserted transperineally and were guided by the placement of a finger in the rectum. In 1972, Whitmore introduced the implantation of iodine-125 seeds into prostatic cancer, and this modality has been receiving increasing interest since then (7).

One of the main disadvantages of brachytherapy is that larger extracapsular tumors with ill-defined margins may not be irradiated adequately; in addition, regional lymphatics may not be irradiated either. Furthermore, with the standard free-hand technique, it is difficult to obtain satisfactory even distribution of the seeds in the gland. Another limitation is that the conventional surgical implantation technique carries inherent risks. Over the years, the procedure for prostate cancer brachytherapy has undergone various modifications. For example, Holm described an ultrasonically guided percutaneous implantation technique in 1983 (7). However, even with this procedure, it is difficult to achieve even distribution of seeds and avoid the risks associated with surgery. In 1990, Holm developed the seed implantation technique further by combining it with transverse and longitudinal scanning (8). In the late 1980s, Ragde from Seattle performed this technique in the USA, and since then, Blasko, Routman, and others have treated numerous patients, refined the technique, taught many courses, and published extensively on this technique (9–12). They have obtained very promising prostate-specific antigen (PSA)-based results that are favorable and comparable with those of radical prostatectomy and external beam radiation for the treatment of prostatic cancer. Thus, there is ample evidence in the literature for the benefits of ultrasound imaging-guided brachytherapy.

Although the implantation of seeds under ultrasound guidance is recognized as a standard treatment, it does have certain drawbacks. For example, between January 1999 and December 2012, a total of 346 medical events related to prostate seed implants were reported to the Nuclear Regulatory Commission or agreement states. Out of the 346 reported medical events, 104 were associated with seed or needle misplacement and led to the administration of excessive doses to normal tissues or organs (13, 14). This type of seed misplacement is an inherent drawback of the standard implant method, but it can be avoided with the use of imaging techniques such as computed tomography (CT). In 1998, Koutrouvelis first reported CT-guided prostate seed implantation. Compared to standard ultrasound-guided brachytherapy, the CT-guided approach allows for the treatment of prostate glands that are larger than 60 cm3 (15). With the CT-guided approach, there is no interference by the pubic arch. Therefore, this method can be used in cases where reduction of prostatic volume with hormone manipulation is not effective. With CT-guided seed implantation in the trans-ischiorectal space, it has been reported that the maximum deviation from the prescribed radiation dose is less than 10% and no seeds are implanted outside the target volume. Further, with high-resolution CT imaging, needles, seeds, and urethra can be identified with a Foley catheter, and this decreases the likelihood of urethral penetration and placement of the seeds in the wall of the urethra and bladder. In addition, the use of a plane template along with CT and magnetic resonance imaging (MRI) to guide particle implantation is an ongoing subject of research (16, 17). However, according to Koutrouvelis’s study on the use of parallel needles, even with these imaging techniques, it is difficult to ensure conformity of the dose cover while simultaneously lowering the dose delivered to the urethra. Furthermore, there are other drawbacks such as the need for a greater number of needles and rectal injuries (15).

With the development of three-dimensional (3D) printing technology, the application of 3D-printed templates for guidance in prostate cancer brachytherapy has come to light. In our previous study, we found that with the help of a 3D-printed template, needles can be accurately inserted into the target according to the preoperative plan and blood vessels and bones can be avoided (18, 19). The distribution of the radioactive seeds was more confined to the tumor, and therefore, this method could provide better compatibility with the dosimetric requirements. After the promising results obtained with the application of the 3D printed template in pelvic cancer, we successfully implanted the radioactive seeds in a patient with prostate cancer to provide a dose boost after radiotherapy under the guidance of a 3D-printed template (20). Following in the success of our previous treatments, in the present study, we report the successful application of 3D printed templates to trans-pararectal iodine-125 seed brachytherapy for prostate cancer in two elderly patients. This is a new technique for the insertion of seeds in brachytherapy for prostate cancer, and we believe the findings from these cases would be useful for the future application of this technique to prostate cancer brachytherapy.

## Case representation

### Case report 1

A 62-year-old male patient with prostate adenocarcinoma (stage cT4N1M0) was admitted to our hospital on June 12, 2018. The patient had previously been pathologically diagnosed with prostate acinar adenocarcinoma (Gleason score, 3 + 4 = 7) in November 2015, and had received radiotherapy and endocrine therapy. The endocrine therapy included bikaluamide tablet (50 mg orally, once a day) and goseraline acetate sustained-release implant (3.6 mg, subcutaneously injected once every 28 d). Two months later, the PSA decreased to 4.4 ng/ml from the baseline level of 12.75 ng/ml. In September 2017, the patient’s PSA was 4.19 ng/ml, and the treatment regimen was changed to oral flutamide 250mg three times a day only. In March 2018, the PSA increased to 10.06 ng/mL, and MRI revealed an enlarged prostate tumor involving the seminal vesicles. The patient received radiotherapy at a prescribed dose of 66 Gy/33 f/2 Gy.CTV: prostate and all seminal vesicles and invaded bladder wall; PTV: CTV is put out for 1cm, and put out for 5mm in the rectum direction. Preventive irradiation of lymph node drainage area (including total iliac, internal iliac, external iliac, obturator, presacral), with the dose of 50Gy/25f, and the dose of 60Gy/30f for external iliac lymph node metastasis.However, after 30 sessions of radiotherapy at a dose of 60 Gy, the patient could not tolerate the rectal side effects of radiotherapy. Therefore, radiotherapy was discontinued. With regard to the history of the patient, he had been diagnosed with coronary atherosclerotic heart disease and anterior myocardial infarction 2 years ago. Digital rectal examination revealed that the texture of the anterior wall of the rectum was slightly hard, and the prostate was hard to touch. The prostate was slightly enlarged, and the boundary of the tumor was not clear. Further, no obvious nodules were palpable. MRI revealed involvement of bilateral seminal vesicles and the posterior wall of the bladder. The serum PSA concentration was 10.16 ng/mL. Anesthesiology evaluation the patient could not tolerate general anesthesia or epidural anesthesia on account of coronary heart disease. Therefore, iodine-125 seed implantation based on a 3D-printed template was performed with the patient under local anesthesia on June 19, 2018. Fifty seeds were implanted, and the preoperative target volumes were as follows: D90: 51.9 Gy, D100: 25.8 Gy, V90: 94.9%, V100: 91.8%, V150: 58.7%; rectum: D2cc: 26 Gy; urethra: D10: 75.2%, D30: 61.8 Gy. Activity was set as 0.3 mCi. The postoperative target volumes were as follows: D90: 53.4 Gy, D100: 32.2 Gy, V90: 96.9%, V100: 93.6%, V150: 60%; rectum: D2cc: 21.4 Gy; urethra: D10: 100.7 Gy, D30: 91.5 Gy. All the dosimetric parameters were consistent with those determined in the preoperative plan. PSA decreased to 6.93 ng/ml at 2 months after the procedure. During the subsequent 3-year follow-up, the PSA concentration was found to be in the normal range. The patient died of myocardial infarction in August 2021 but there was no recurrence of prostate cancer locally.

### Preoperative preparation

**Figure 1:**
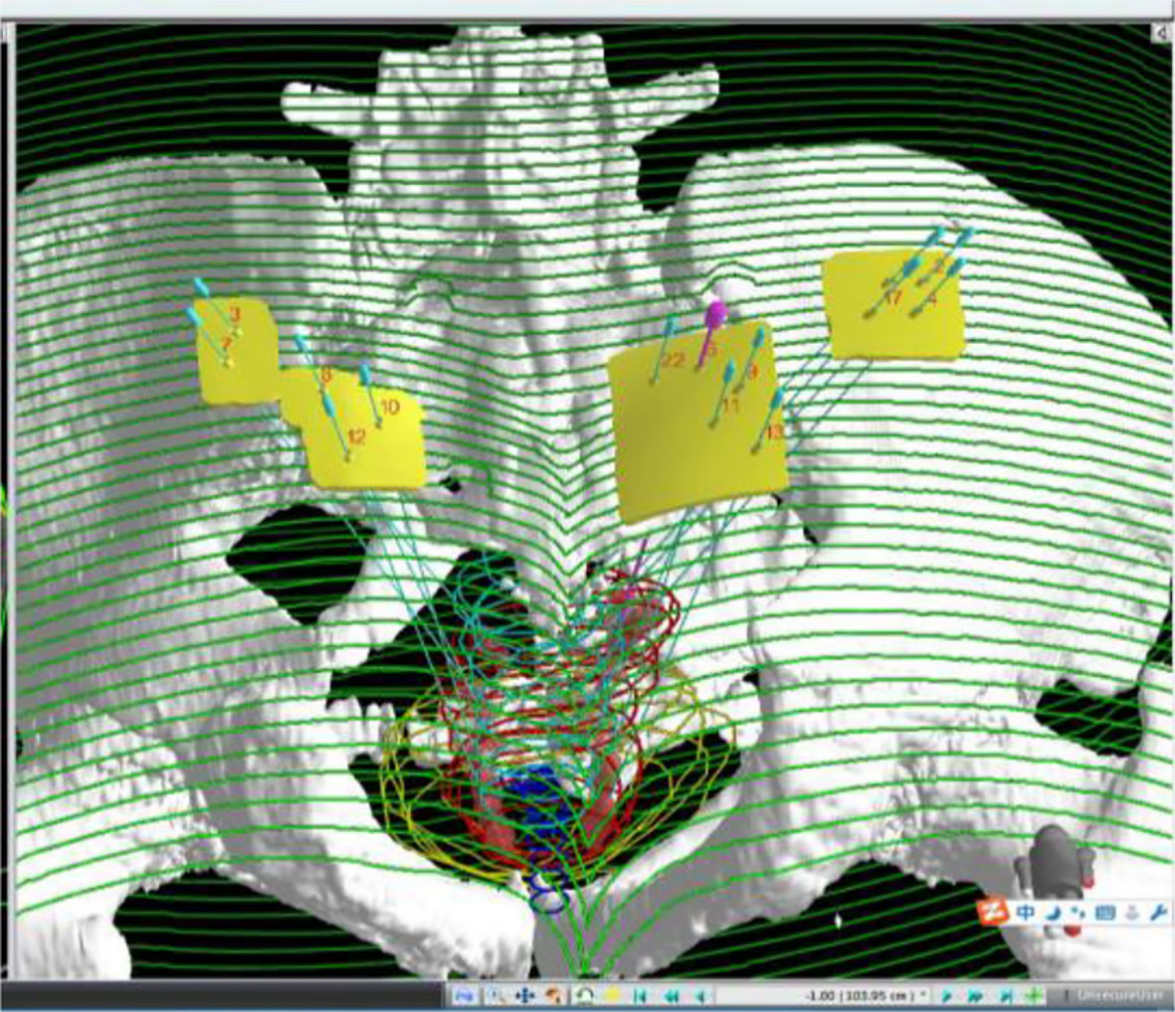
Three-dimensional reconstruction of targets, organs at risk, needle channels, and templates on TPS.

**Figure 2:**
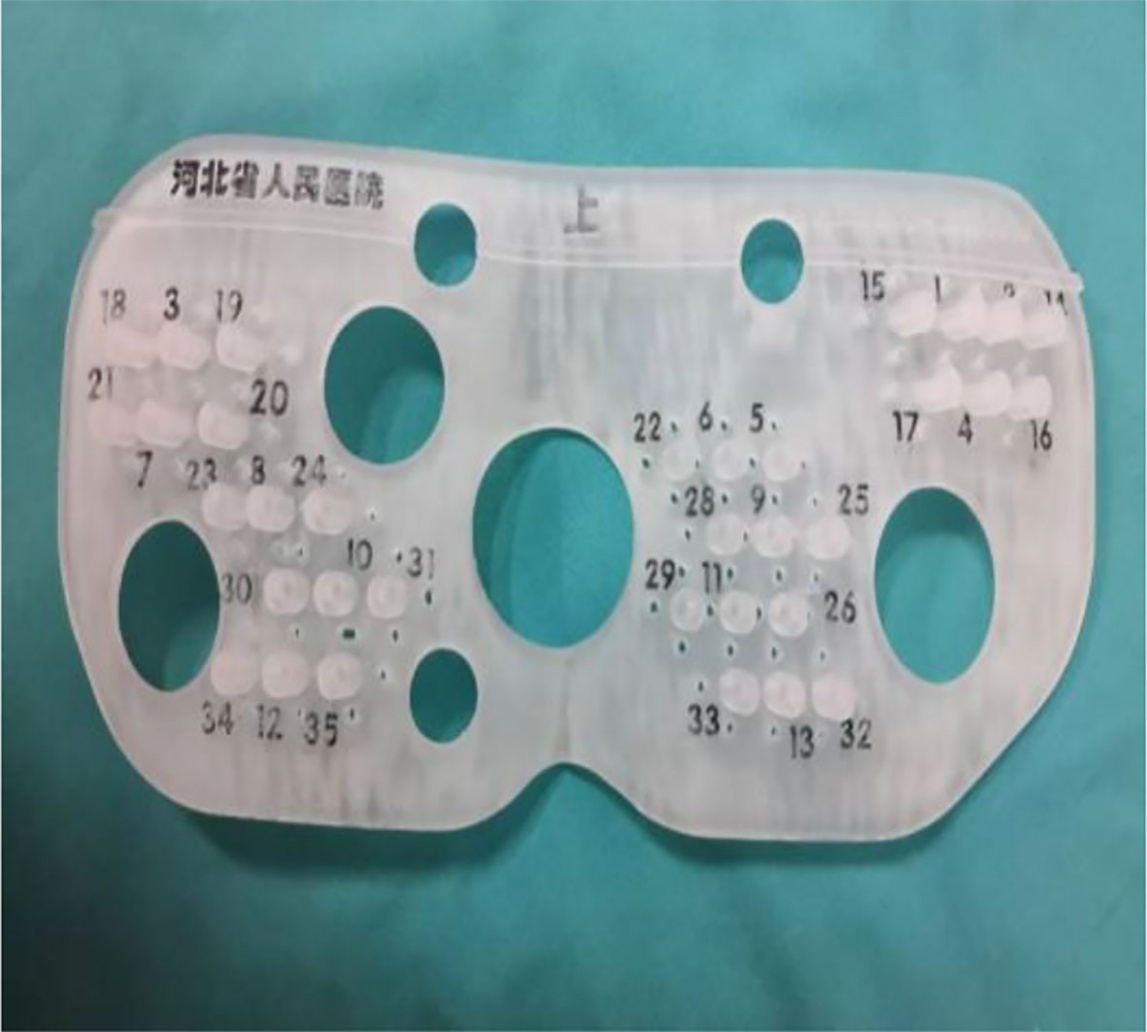
Design and print of an individualized 3D template according to the preoperative plan.

One week before the implantation procedure, the patients were immobilized in a prone position with a vacuum cushion. A position line was drawn using a CT laser on the skin of the buttocks, and two marks were made 3–4 cm away on this line. An enhanced CT scan was performed in order to obtain an image series with a slice interval of 5 mm. The preoperative plan was designed by the physicist with Panther Brachy version 5.0 TPS. The number and distribution of seeds required were calculated to deliver a minimum peripheral tumor dose of 145 Gy. The surface of the patient’s buttocks, the template to be printed, and needle coordinates were reconstructed in Prowess TPS (**Figure 1**). Following this, the 3D printing output file was generated and the 3D template was printed (**Figure 2**). One day before implantation, the 3D-printed template was disinfected and sterilized.

### Procedure

**Figure 3:**
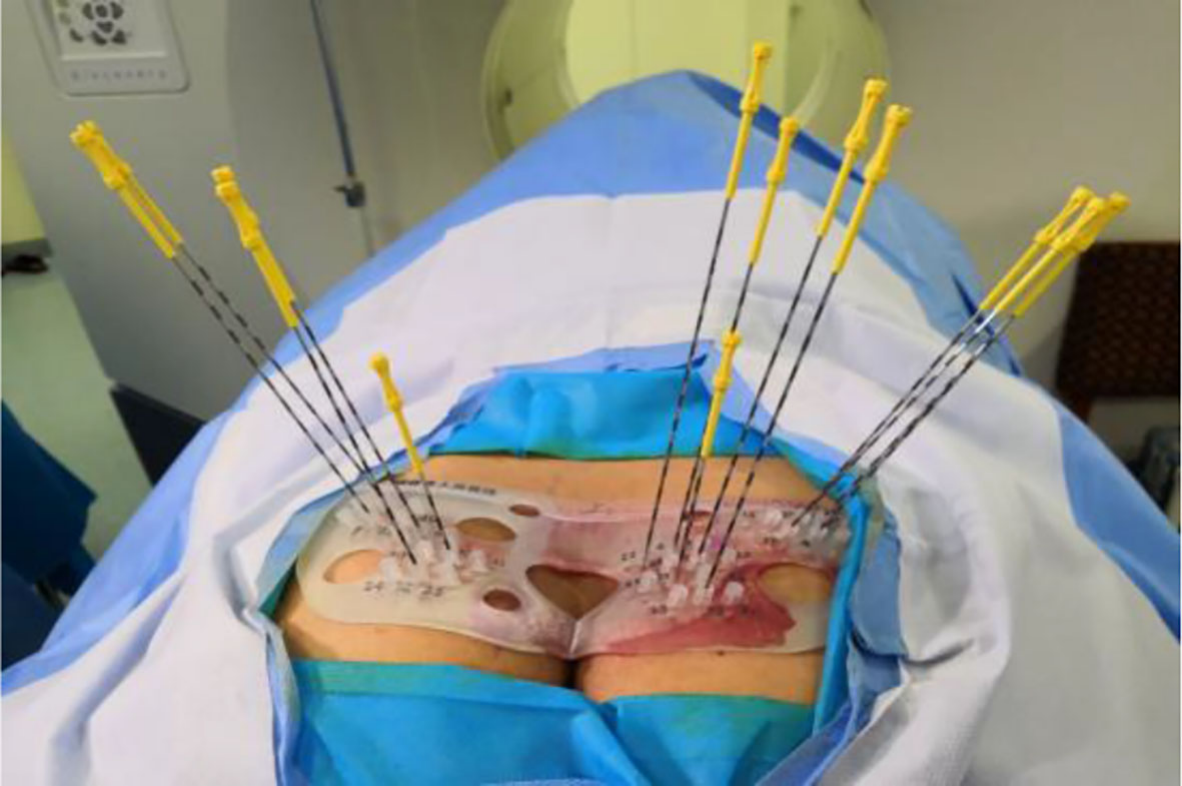
Setting of the template and insertion of the particle needles.

**Figure 4:**
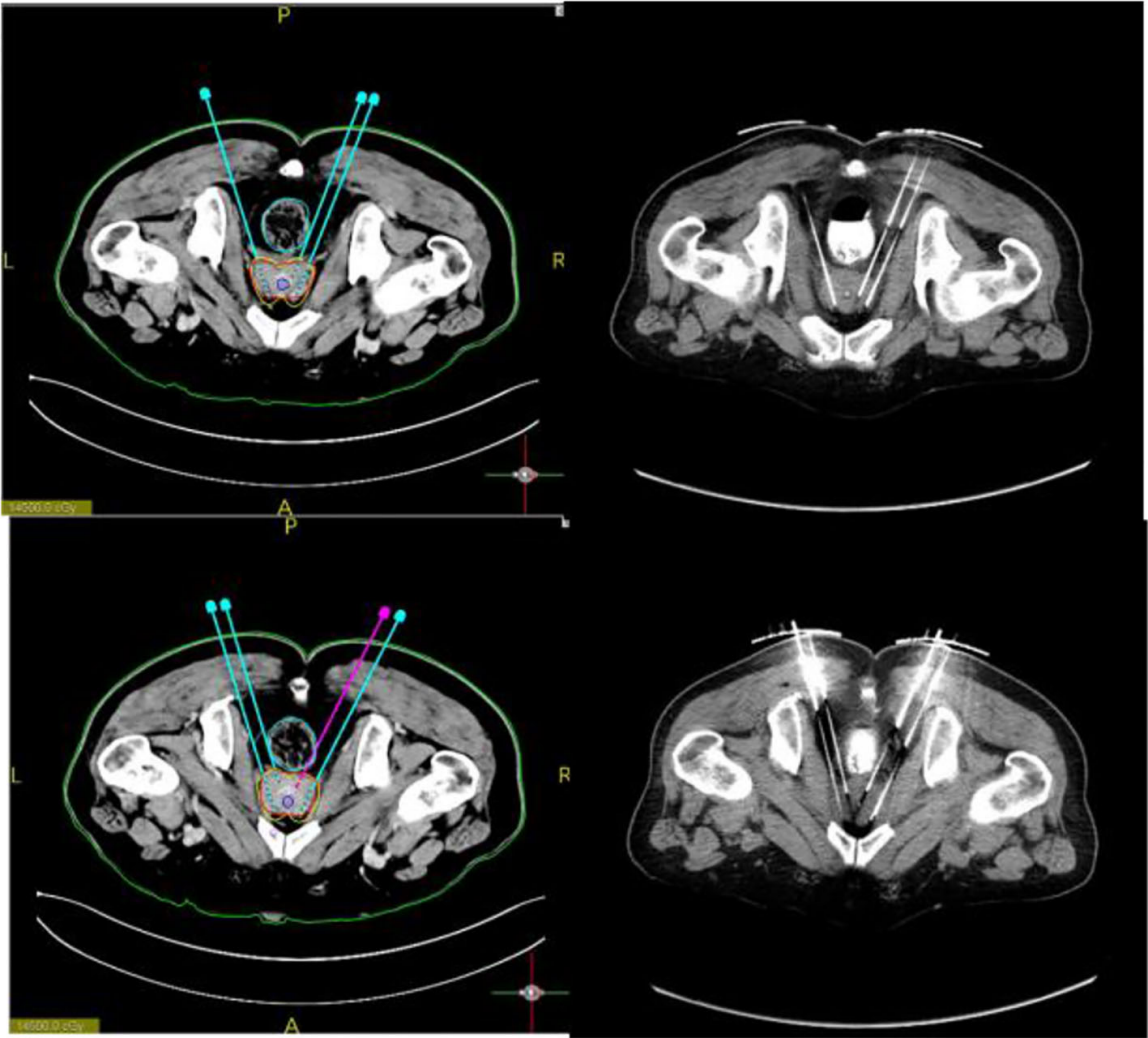
Preoperative design. The needle path is planned and particles are loaded to calculate the isometric line distribution. Further, the puncture needle path was accurately implemented during the procedure.

**Figure 5:**
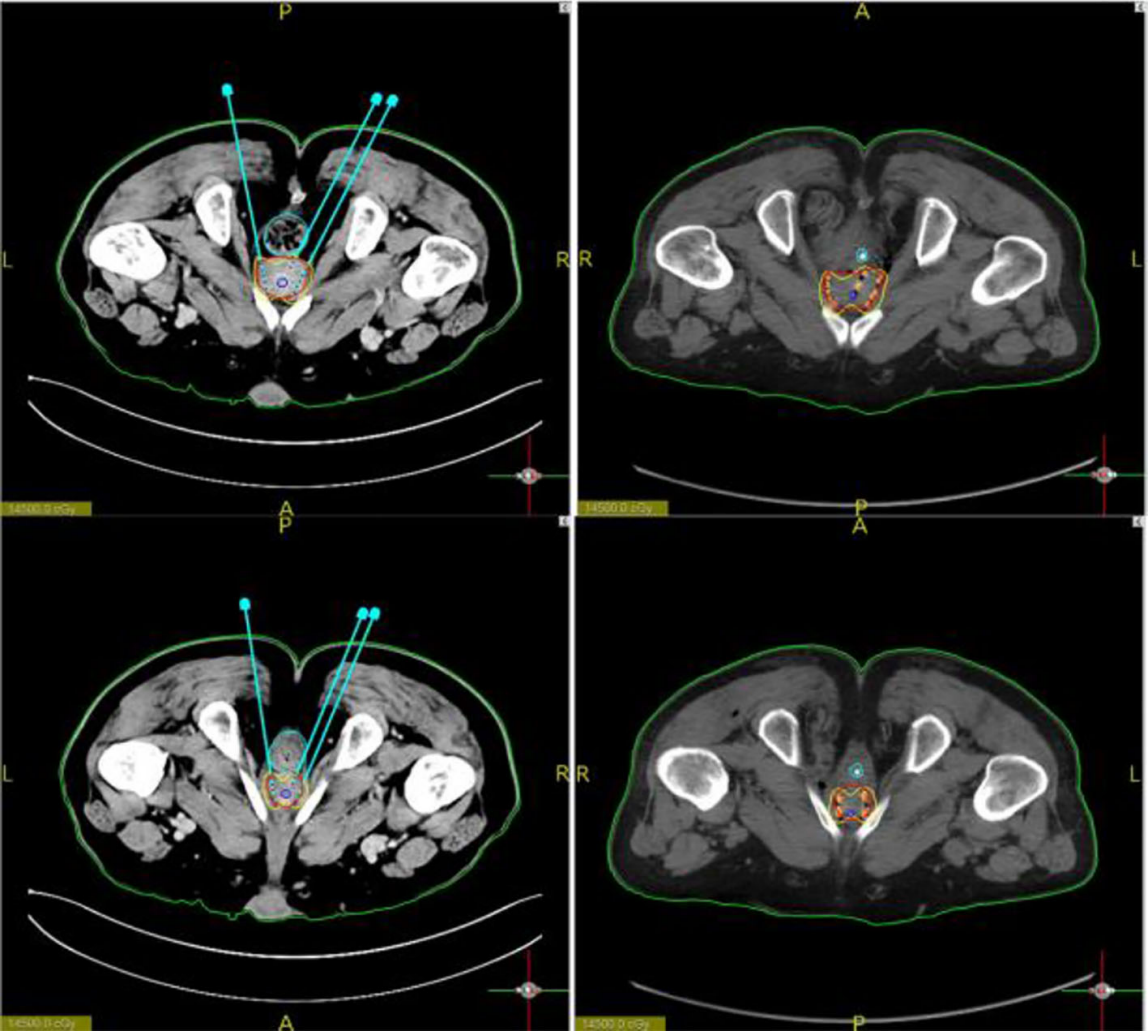
Images showing particle positions. Particle positions according to the preoperative plan and the postoperative image basically coincided.

**Figure 6:**
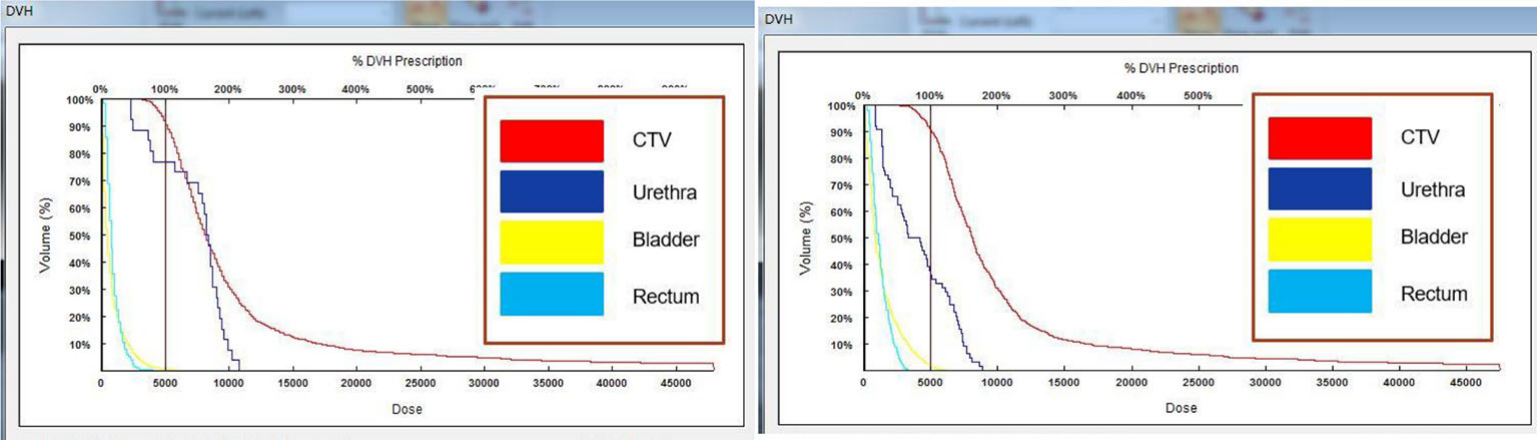
DVH plots before and after surgery. Comparison of the plots indicated that the postoperative dose parameters met the requirements of the preoperative plan.

During the operation, the vacuum cushion was used to fix the patient in the prone position used during the preoperative planning (by referring to the photograph taken of the patient in the immobilized position). The CT laser line was aligned with the line drawn on the surface of the patient’s skin preoperatively. After sterilization of the skin, the disinfected 3D-printed template was secured on the patient’s body surface based on the markers attached. A CT scan was performed to confirm that the template position was correct, and then the needles were fed into the tumor target based on the position of the template holes (**Figure 3**). After all the needles were inserted, a CT scan was performed again to confirm the position of the needles (**Figure 4**). After it was confirmed that all the needles were in the right position according to the preoperative plan, the radioactive seeds were implanted. Postoperative verification of the dose was also performed with CT (**Figure 5**). DVH was performed after operation and compared with that before operation (**Figure 6**).

### Case report 2

A 75-year-old male patient who presented with prostate adenocarcinoma (stage cT2aN0M0) was admitted to our hospital on May 31, 2018. The pathological diagnosis was prostate acinar adenocarcinoma (Gleason score, 3 + 4 = 7), and the PSA values were as follows: total PSA, 11.99 ng/m1; free PSA, 1.55 ng/mL; free/total PSA, 0.13. The patient had a history of Grade III hypertension (blood pressure, up to 200/100 mm Hg) for more than 7 years. Digital rectal examination revealed an enlarged prostate (degree I), a palpable fovea, hard texture of the right side of the lobe, and palpable small nodules. MRI examination demonstrated a central prostate lobe nodule that carried the possibility of prostate cancer. The patient was in the medium-risk group for prostate cancer but refused surgery and external radiotherapy. Therefore, iodine-125 seed implantation with the help of a 3D-printed template was performed under local anesthesia on June 28, 2018, and 58 seeds were implanted. The preoperative target volumes were as follows: D90: 148 Gy, D100: 88.5 Gy, V90: 94.6%, V100: 91.5%, and V150: 49.1%. Activity was set as 0.45 mCi. The postoperative target volumes were as follows: D90: 150 Gy, D100: 99.9 Gy, V90: 96.4%, V100: 91.7%, and V150: 51% (**Figure 6**). All the parameters were consistent with the preoperative plan, and the PSA values at 1 month after the implantation were as follows: total PSA, 0.63 ng/m1; free PSA, 0.102 ng/mL; free/total PSA, 0.16. The patient continued to take bicalutamide 50mg once a day regularly 6 months after the procedure. After that, the patient stopped taking medicine by himself, During the subsequent 4-year follow-up, the PSA concentrations remained in the normal range. The patient is still alive.

## Results

**Figure 7:**
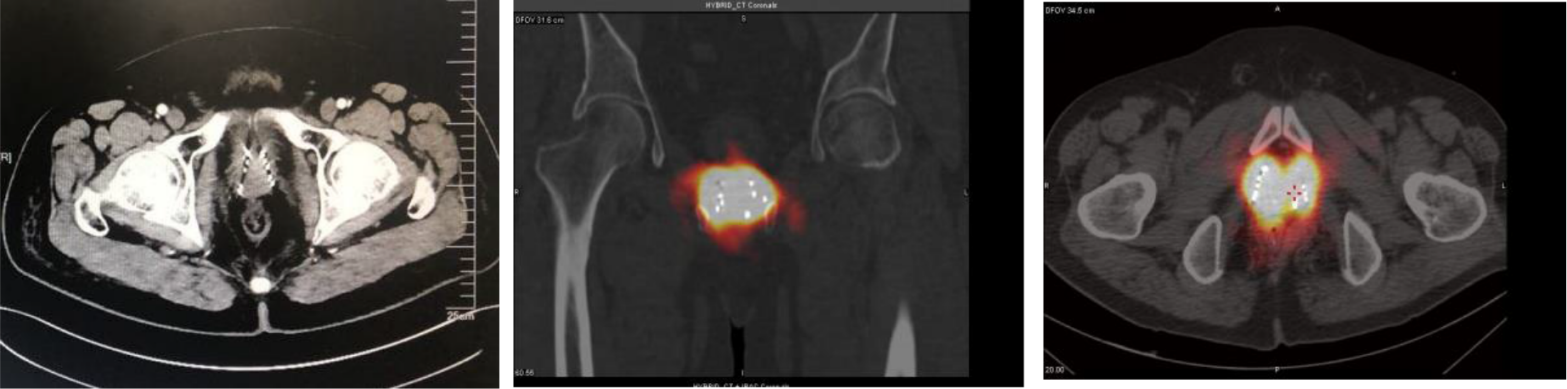
After 1 month of re-examination, no change was observed in tumor size, and SPECT showed that the radioactive concentration of particles basically covered the target area.

The procedure was successfully completed in the two cases described above. In one case, the prostate volume was 62.8 cm3, and 50 seeds were implanted by accurate positioning of 13 needles into the prostate. In the second case, the prostate volume was 33.9 cc, and 58 seeds were implanted by accurate insertion of 14 needles. Slight soft tissue hemorrhage was noted in one case, but it resolved spontaneously without the need for special treatment. Hematuria was detected in the second patient, but it disappeared on the third day after the procedure. No seed migration or passing of seeds was observed. As of now, the patients have been followed up for 50 months, and their PSA levels have been normal over this follow-up period. Further, no local recurrence has occurred, and SPECT showed that the radioactive concentration of particles basically covered the target area. (**Figure 7**)

## Discussion

The aim of our case report is to describe a new technique for the insertion of seeds in brachytherapy for patients with prostate cancer. In both cases, brachytherapy was successfully completed with the technique using a 3D-printed template under CT guidance. Only a couple of minor complications occurred, but they resolved spontaneously without the need for additional treatment. The patients’ PSA levels were normal at the last follow-up, which was conducted 50 months after the procedure. Importantly, postoperative examination confirmed that the needles were accurately placed according to the preoperative plan, and the postoperative and preoperative dosimetric parameters showed correspondence.

In our previous study conducted in 2015, 3D printed template was combined with seed implantation for the first time, and our findings were reported in Chinese for the first time in March 2016^[19]^. In this study, a patient with recurrent rectal cancer near the prostate successfully underwent seed implantation under the guidance of a 3D printed template through the perirectal pathway. With the guidance of the 3D printed template, the needle can be inserted into the tumor at any angle while simultaneously avoiding injury to normal tissues. After 2016, many publications have demonstrated the accuracy of needle insertion under the guidance of the 3D printed template (21–24). Based on these published findings, we used this technique to treat the present two cases of prostate cancer in which radiotherapy could not be completed because of rectal complications. Based on the findings of the present cases and previously reported ones, the advantages of this technique over the standard procedure for seed implantation in the prostate are as follows. (1) Patients can undergo the procedure in the prone position, which is comfortable and lowers the risk of thrombosis of the lower limb. (2) Patients with heart disease who have intolerance towards general anesthesia can be treated under local anesthesia. (3) The time-consuming preoperative plan is completed before the procedure, so the operation time is considerably shorter. (4) Implantation of seeds outside of the prostate can be avoided under CT guidance. (5) As the number of needles required is reduced, the risk of complications associated with needle insertion is reduced. (6) Seeds can be inserted more precisely and distributed more evenly than with the standard technique. (7) The preoperative plan can optimize the dose during the procedure and, thereby, guarantee adequate dose distribution. (8) Postoperative dose verification can be completed before the needles are pulled out, and more seeds can be implanted immediately if the dose is not sufficient. (9) Prostate tumors of large volume can be treated. Despite these evident advantages of the technique, more clinical research is required to verify whether this procedure can improve the results of brachytherapy for prostate cancer.

Although there are still some issues with this new method, such as the time-consuming preoperative planning and design phase, the skills required for 3D printed template reset, and needle path deviation due to the long puncture path, any new technique requires a learning curve. Even with the above disadvantages, this procedure has obvious advantages over the traditional one. First, the indications for this procedure are extensive, and it can be applied to a wide variety of cases. Second, the use of local anesthesia is beneficial for patients with abnormal cardiopulmonary function and provides better intraoperative comfort. Third, this method improves surgical efficiency, eliminates the need for preoperative preparation under general anesthesia and special intestinal preparation, simplifies the surgical process, considerably shortens the surgical time, and can be completed by any physician with experience in and knowledge about puncture and CT imaging. In addition, as the needle passages are planned in advance, organs such as blood vessels and intestines can be avoided. After template reduction and fixation, the tumor can be punctured accurately according to the preoperative plan. In addition, the number of needle passages required is less than that required with the traditional method, so the amount of collateral damage associated with this procedure is low. Thus, overall, this method seems to be more advantageous than disadvantageous and warrants further research and application in the future to confirm the benefits reported in the cases so far, including the present cases.

## Data availability statement

The original contributions presented in the study are included in the article/Supplementary Material. Further inquiries can be directed to the corresponding author.

## Ethics statement

Written informed consent was obtained from the individual(s) for the publication of any potentially identifiable images or data included in this article.

## Author contributions

XD and HZ conceived of the study, and participated in its design and coordination and helped to draft the manuscript. XL, JZ and ZG searched the paper. HY, XS and YL collected data. JW revised the manuscript. All authors contributed to the article and approved the submitted version.

## Conflict of interest

The authors declare that the research was conducted in the absence of any commercial or financial relationships that could be construed as a potential conflict of interest.

## Publisher’s Note

All claims expressed in this article are slely those of the authors and do not necessarily represent those of their affiliated organizations, or those of the publisher, the editors and the reviewers. Any product that may be evaluated in this article, or claim that may be made by its manufacturer, is not guaranteed or endorsed by the publisher.

## References

[1] Holm HH. The history of interstitial brachytherapy of prostatic cancer. Semin SurgOncol (1997) 13(6):43137. doi: 10.1002/(sici)10982388(199711/12)13:6<431:aid-ssu7>3.0.co;2-b 9358590

[2] Albert M Song JS Schultz D. Defining the rectal dose constraint for permanent radioactive seed implantation of the prostate. Urol Oncol-Seminori (2008) 26(2):147–52. doi: 10.1016/j.urolonc.2007.03.026 18312933

[3] Muraki K Hattori C Ogo E. Analysis of radioactive implant migration in patients treated with iodine-125 seeds for permanent prostate brachytherapy with MRI-classified median lobe hyperplasia. Etsuyo OgoJ Contemp Brachytherapy (2021) 13(3):254–62. doi: 10.5114/jcb.2021.105944 PMC8170528 34122564

[4] Okamoto K. Ten-step method of high-dose LDR 125 I brachytherapy for intermediate-risk prostate cancer. J Appl Clin Med Phys (2021) 22(6):172–82. doi: 10.1002/acm2.13224 PMC8200501 33939267

[5] Zelefsky MJ Moughan J Owen J. Changing trends in national practice for external beam radiotherapy for clinically localized prostate cancer: 1999 patterns of care survey for prostate cancer. Int J Radiat Oncol Biol Phys (2004) 59:1053–61. doi: 10.1016/j.ijrobp.2003.12.011.:10.5414/CN109582 15234039

[6] Barringer BS. Radium in the treatment of carcinom a of the bladder and prostate. JAMA (1917) LXVIII(17):1227. doi: 10.1001/jama.1917.04270040215002

[7] Whitmore WF Jr Hilaris B Grabstald H. Retropubic implantation of iodine 125 in the treatment of prostatic cancer. J Urol (1972) 108(6):918–20. doi: 10.1016/s0022-5347(17)60906-6 5082747

[8] Holm HH Myschetzky P Nielsen L Nolsoe C. An ultrasonic multipurpose/multiplane endoprobe. Acta Radiol (1990) 31(6):630–3. doi: 10.1080/02841859009173114 2278794

[9] Blasko JC Wallner K Grimm PD Ragde H. Prostate specific antigen based disease control following ultrasound guided 125 iodine implantation for stage T1/T2 prostatic carcinoma. J Urol (1995) 154(3):1096–9. doi: 10.1097/00005392-199509000-00052 7543606

[10] Routman DM Funk RK Stish BJ Mynderse LA Wilson TM McLaren R. Permanent prostate brachytherapy monotherapy with I-125 for low- and intermediate-risk prostate cancer: Outcomes in 974 patients. Brachytherapy (2019) 18(1):1–7. doi: 10.1016/j.brachy.2018.09.003 30293836

[11] Anderson E Smyth LML O’Sullivan R Ryan A Lawrentschuk N Grummet J. Focal low dose-rate brachytherapy for low to intermediate risk prostate cancer: Preliminary experience at an Australian institution. Transl Androl Urol (2021) 10(9):3591–603. doi: 10.21037/tau-21-508 PMC8511546 34733655

[12] Smile TD Tom MC Halima A Ciezki JP Reddy CA Stephans KL. 125 I interstitial brachytherapy with or without androgen deprivation therapy among unfavorable-intermediate and high-risk prostate cancer. Brachytherapy (2022) 21(1):85–93. doi: 10.1016/j.brachy.2021.09.001 34656435

[13] Chief PP. (2010). Multiple medicalevents involving prostate brachytherapy treatments at department of veterans affairs medical centerphiladelphia-update, in: ACMUI Meeting, Washington, DC.

[14] Gao WB. Analysis on the reporting of medical events in permanent prostate brachytherapy. Cureus (2013). doi: 10.7759/cureus.133

[15] Koutrouvelis PG. Three -dimensional stereotactic posterior ischiorectalspace computerized tomography guided brachytherapy of prostatecancer: A preliminary report. J Urol (1998) 159(1):142–5. doi: 10.1016/s0022-5347(01)64037-0 9400457

[16] Chatterjee A Turchan WT Fan X. Can pre-treatment quantitative multi-parametric MRI predict the outcome of radiotherapy in patients with prostate cancer? Acadrdiol (2022) 29(7):977–85. doi: 10.1016/j.acra.2021.09.012 34645572

[17] Wang J Zhang F Guo J. Expert consensus workshop report: Guideline for three-dimensional printing template-assisted computed tomography-guided 125I seeds interstitial implantation brachytherapy. J Cancer Resther (2017) 13(4):607–12. doi: 10.4103/jcrt.JCRT_412_17 28901301

[18] Zhang HT Di XM Yu HM. Dosimetry study of three-dimensional print template-guided precision 125Iseed implantation. J Cancer Res Ther (2016) 12(Suppl):C159–65. doi: 10.4103/0973-1482.200607 28230010

[19] Liang YS Wang ZY Zhang HT. T Three-dimensional-printed individual template-guided 125 I seed implantation for the cervical lymph node metastasis: A dosimetric and security study. J Cancer Res Ther (2018) 14(1):30–5. doi: 10.4103/jcrt.JCRT_619_17 29516955

[20] Zhang HT Di XM Yu HM. Dose comparison between pre and post operation of 125I seeds implantation guided by 3D print template. Chin Med J (2016) 96(9):712–5. doi: 10.3760/cma.j.issn.0376-2491.2016.09.010 27055510

[21] Gao Z Zhang HT Zhang LJ. 3D-printed template-guided 125I seed brachytherapy: A salvage approach for locoregional refractory recurrence of papillary thyroid cancer. Eur Thyroid J (2021) 10(6):504–10. doi: 10.1159/000519572 PMC8647112 34950601

[22] Han X Fang S Sheng R. Dosimetry verification of three-dimensional printed polylactic acid template-guided precision 125 I seed implantation for lung cancer using a desktop three-dimensional printer. J Appl Clin Med Phys (2021) 22(10):202–9. doi: 10.1002/acm2.13419 PMC8504606 34487634

[23] Qu A Jiang P Wei S. Accuracy and dosimetric parameters comparison of 3D-printed non-coplanar template-assisted computed tomography-guided iodine-125 seed ablative brachytherapy in pelvic lateral recurrence of gynecological carcinomas. J Contemp Brachyther (2021) 13(1):39–45. doi: 10.5114/jcb.2021.103585 PMC8117710 34025735

[24] Wang H Peng R Li X. The dosimetry evaluation of 3D printing non-coplanar template-assisted CT-guided 125I seed stereotactic ablation brachytherapy for pelvic recurrent rectal cancer after external beam radiotherapy. J Radiat Res (2021) 62(3):473–82. doi: 10.1093/jrr/rraa144 PMC8127672 33616168

